# Effects of speech rate modifications on phonatory acoustic outcomes in Parkinson’s disease

**DOI:** 10.3389/fnhum.2024.1331816

**Published:** 2024-02-21

**Authors:** Thea Knowles, Scott G. Adams, Mandar Jog

**Affiliations:** ^1^Department of Communicative Sciences and Disorders, Michigan State University, East Lansing, MI, United States; ^2^School of Communication Sciences and Disorders, Western University, London, ON, Canada; ^3^Health and Rehabilitation Sciences, Western University, London, ON, Canada; ^4^Department of Clinical Neurological Sciences, University Hospital, London, ON, Canada

**Keywords:** Parkinson’s disease, deep brain stimulation, speech rate control, speech acoustics, aging

## Abstract

Speech rate reduction is a global speech therapy approach for speech deficits in Parkinson’s disease (PD) that has the potential to result in changes across multiple speech subsystems. While the overall goal of rate reduction is usually improvements in speech intelligibility, not all people with PD benefit from this approach. Speech rate is often targeted as a means of improving articulatory precision, though less is known about rate-induced changes in other speech subsystems that could help or hinder communication. The purpose of this study was to quantify phonatory changes associated with speech rate modification across a broad range of speech rates from very slow to very fast in talkers with and without PD. Four speaker groups participated: younger and older healthy controls, and people with PD with and without deep brain stimulation of the subthalamic nucleus (STN-DBS). Talkers read aloud standardized sentences at 7 speech rates elicited using magnitude production: habitual, three slower rates, and three faster rates. Acoustic measures of speech intensity, cepstral peak prominence, and fundamental frequency were measured as a function of speech rate and group. Overall, slower rates of speech were associated with differential effects on phonation across the four groups. While all talkers spoke at a lower pitch in slow speech, younger talkers showed increases in speech intensity and cepstral peak prominence, while talkers with PD and STN-DBS showed the reverse pattern. Talkers with PD without STN-DBS and older healthy controls behaved in between these two extremes. At faster rates, all groups uniformly demonstrated increases in cepstral peak prominence. While speech rate reductions are intended to promote positive changes in articulation to compensate for speech deficits in dysarthria, the present results highlight that undesirable changes may be invoked across other subsystems, such as at the laryngeal level. In particular, talkers with STN-DBS, who often demonstrate speech deterioration following DBS surgery, demonstrated more phonatory detriments at slowed speech rates. Findings have implications for speech rate candidacy considerations and speech motor control processes in PD.

## 1 Background and rationale

The majority of individuals with Parkinson’s disease (PD) will develop hypokinetic dysarthria (HkD) at some point during the course of the disease (Logemann et al., 1978; Mutch et al., 1986; Müller et al., 2001). The most prominent speech features of HkD have led to its characterization of prosodic insufficiency. Auditory-perceptual features include reduced speech loudness, monotone and monoloud prosody, abnormal rates of speech, including fast rushes of speech, and imprecise articulation (Darley et al., 1969a,b). Of these, phonatory impairments tend to be the most frequently occurring and perceptually salient (Logemann et al., 1978; Ludlow et al., 1987) and are often detectable even in early, mild stages (Skodda et al., 2013) and in prodromal disease stages (Rusz et al., 2011, 2016). Functionally, speech and voice changes in PD can lead to difficulties in being understood and a subsequent reduction in overall communicative quality of life. Gold-standard treatment approaches aimed at improving communication in individuals with PD are those that are considered global speech treatments. Global, in contrast with system-specific speech treatments, target compensation for a singular speech feature, such as loudness or rate, that results in change across multiple speech subsystems (e.g., articulatory, phonatory, respiratory). Common global treatment approaches for PD include those with a focus on loudness, rate, clarity, or prosody (Yorkston et al., 2007; Tjaden, 2008). Here, we focus on adjustments to one such approach, speaking rate, and evaluate its consequences on phonatory impairments. This work extends our previous investigations of the effects of speech rate modification in two groups of talkers with PD by introducing the consequences of rate modification on phonatory acoustics.

### 1.1 Speech symptoms and acoustic correlates in PD

Phonatory abnormalities have been reported in up to ∼90% of individuals with PD at some point during the course of the disease (Logemann et al., 1978). Perceptually, voice symptoms include a quiet, hoarse quality marked by monoloudness and monotone (Darley et al., 1969a). Acoustically, speech in PD is often marked by low speech intensity (Fox and Ramig, 1997; Ho et al., 1999; Adams et al., 2005) and increased noise in the signal (Ramig et al., 1988; Zwirner and Barnes, 1992; Hertrich and Ackermann, 1995; Gamboa et al., 1997; Kent and Kim, 2003; Rusz et al., 2011; Cushnie-Sparrow et al., 2018) compared to neurologically healthy age-matched peers. These phonatory symptoms are collectively referred to as hypophonia (Adams and Dykstra, 2009).

Speech in PD is also characterized by abnormal and variable rates of speech. While some people with PD may exhibit slower connected speech rates (Martínez-Sánchez et al., 2016; Hsu et al., 2017), others may produce faster rates of speech, a unique symptom among the dysarthrias (Darley et al., 1969a). Acceleration of speech rate has also been reported in PD (for example, over the course of reading a passage), even in the absence of overall group differences in speech rate (Adams, 1994; Skodda and Schlegel, 2008) or syllable repetition (Netsell et al., 1975; Hirose et al., 1982; Ackermann et al., 1995; Skodda, 2011). In a review of speech symptoms reported in PD, Adams and Dykstra (2009) suggested a prevalence of abnormally fast rates of approximately 6 to 13%. As such, fast rates may not often be evident at the group level, but may manifest in a subset of people with PD.

Deep brain stimulation of the subthalamic nucleus (STN-DBS) is an increasingly common adjunctive surgery for the gross motor symptoms of PD, typically recommended for individuals who have developed adverse motor fluctuations and side effects to the standard pharmaceutical treatment (Limousin et al., 1998; Okun, 2012). Reports of speech changes following STN-DBS surgery suggest tremendous variability in individual outcomes (Aldridge et al., 2016). Some studies have shown relative improvements in speech intensity (Lundgren et al., 2011), while others have shown declines (Dromey et al., 2000). Reports are similarly inconsistent regarding changes in measures of vocal perturbation (Gentil et al., 2001, 2003; D’Alatri et al., 2008; Putzer et al., 2008; Sidtis et al., 2010; Dromey and Bjarnason, 2011; Martel-Sauvageau et al., 2015; Tanaka et al., 2015; Tsuboi et al., 2015) as well as in speech rate (Wang et al., 2006; Klostermann et al., 2008; Karlsson et al., 2011; Eklund et al., 2014; Tripoliti et al., 2014).

### 1.2 Rate reduction

Producing speech at a slower rate has long been targeted as a behavioral intervention for improving speech intelligibility in dysarthria (Yorkston et al., 1990, 2007; Duffy, 2013), including in PD. People with PD may be especially likely to benefit from rate reduction given the prevalence of fast speaking rates unlikely to be seen in other dysarthrias. Speech rate modification is considered a global therapeutic variable because it has the potential to demonstrate effects across multiple speech systems including articulation, respiration, and phonation (Dromey and Ramig, 1998; Yorkston et al., 2007). Early treatment studies found promising links between slower rates of speech and speech severity for some people with PD in case studies or small speaker groups (Downie et al., 1981; Yorkston and Beukelman, 1981; Hanson and Metter, 1983; Caligiuri, 1989; Yorkston et al., 1990).

The majority of studies that have reported on the acoustic consequences of modified speech rate in PD, however, have tended to focus on segmental enhancements. In general, findings have demonstrated that slower speech is associated with increases in vowel space in PD (McRae et al., 2002; Tjaden and Wilding, 2004; Tjaden et al., 2005). A limited body of research suggests that increases in speech intensity, for example, are on the order of ∼1 dB sound pressure level (SPL) in slow speech in PD (Tjaden and Wilding, 2004). Slow speech in talkers with PD has also been associated with, perhaps unexpectedly, decreases in *f*_0_ mean, maximum, and range (Tjaden and Wilding, 2011a). Given that PD is associated with an already reduced baseline for phonatory and prosodic variation, rate reduction may not be ideal for some talkers who exhibit these unintended consequences while speaking.

There are additional reasons to be cautious of anticipating improvements in speech outcomes following rate reductions across the board for people with PD, however. One reason for this is that while some individuals may improve, several studies have reported that some talkers with PD do not exhibit increases in intelligibility when producing slower rates of speech, and some may even worsen (Van Nuffelen et al., 2009, 2010; Hall, 2013; Kuo et al., 2014; Fletcher et al., 2017; McAuliffe et al., 2017). Conversely, while faster speech is not likely to be a treatment target, a small body of literature has demonstrated that intentional increases in speech rate is not necessarily associated with what might be an expected decrease in intelligibility (Kuo et al., 2014), and may even be associated with increases in naturalness or acceptability in some cases (Logan et al., 2002; Dagenais et al., 2006; Sussman and Tjaden, 2012; Kim and Seong, 2015). A further consideration is that natural changes to speech rate occur as a result of typical, healthy aging. In particular, older talkers tend to speak at slower rates than younger talkers (e.g., Jacewicz et al., 2009). Furthermore, there is not a direct relationship between typical speaking rate and speech intelligibility in neurotypical talkers. That is, people with naturally slower speech are not necessarily more (or less) intelligible than those with naturally faster speech (Bradlow et al., 1996).

Yorkston et al. (1999) described the likelihood of a trade-off between speech accuracy and speech naturalness such that, for a given speaker with dysarthria, the there may exist an intelligibility peak. Speaking too slowly in relation to this hypothetical peak would result in poorer understanding because of compromised speech naturalness, whereas speaking too quickly would lead to imprecise articulation. Yorkston et al. (1999) asserted that the goal of speech rate modification intervention is to identify a target rate that “will allow an optimal level of intelligibility without degrading naturalness unnecessarily” (pp. 416). A challenge with existing research is that the majority of studies exploring speech rate modifications in PD have explored a single rate adjustment (e.g., slower), while some have explored a single adjustment in either direction (e.g., slower and faster). More rate adjustments, from very slow to very fast, may provide more detailed insights into the mechanisms that different talkers employ, and how these may impact treatment recommendations. The current study presents extensions from a larger project that investigated acoustic and perceptual consequences of rate modifications across seven speech rate modifications from very slow to very fast in people with PD with and without STN-DBS, as well as with neurologically healthy controls.

### 1.3 Summary and purpose

In order to better understand the effects of speaking rate, more descriptions of multisystem changes are needed across a broader range of speech rates. More detailed descriptions of what individuals do when modifying their rate of speech would help aid identifying existing individual strengths as well as potential maladaptive behaviors that may arise when an individual attempts to implement a modified rate. A descriptive model of speech rate changes would thus better serve to identify candidates and strategies for more effective implementation of rate modification (Turner and Weismer, 1993; Tjaden and Wilding, 2011b). An open question regarding rate modification is the unintended acoustic changes that occur at a phonatory-prosodic level in PD. A better understanding of system-wide changes that occur in speech when individuals modify their rates of speech would not only help inform treatment decisions, but provide insight into mechanisms of motor control during common behavioral intervention practices. The purpose of this study was to quantify the changes made to acoustic measures of voice quality in two groups of individuals with PD and neurologically healthy controls as they modified their rate of speech from very slow to very fast. The following research questions were of interest:

How do changes in speech rates from very slow to very fast affect acoustic phonatory outcomes in:

1. Younger and older talkers? We hypothesize that age-related phonatory changes will be reflected across the speech rate adjustments.
2. People with PD compared to neurologically healthy age-matched controls? We hypothesize that both slower and faster speech rates will cause increases in speech intensity, and decreases in acoustic correlates of voice quality reflecting increased noise in the signal in both groups.
3. People with PD who have undergone STN-DBS surgery compared to those with PD undergoing typical levodopa management? We hypothesize that the two PD groups will behave similarly to each other, but greater variability will be observed in the PD-DBS group.

## 2 Materials and methods

The study was approved by the Health Sciences Research Ethics Board at Western University and the Lawson Health Research Institute.

### 2.1 Participants

Four speaker groups participated: (1) younger healthy controls under 35 years of age (YC; *n* = 17; 9 male, 8 female), (2) older neurologically healthy controls (*n* = 17, 11 male and 6 female, 56–82 years of age), (3) people with PD receiving standard pharmaceutical (levodopa) treatment (PD-Med; *n* = 22, 18 male and 4 female, 56–90 years of age), and (4) people with PD who had received deep brain stimulation of the subthalamic nucleus (PD-DBS; *n* = 13, 11 male and 2 female, 55–72 years of age); PD participants are described in Tables 1, 2. These participants and speech outcomes related to speech intelligibility and stop and vowel articulation have previously been described elsewhere (Knowles et al., 2021a,b).

**TABLE 1:** Participant demographics for the PD-Med speaker group.

ID	Sex	Age	MoCA	Years post-diagnosis	PD medications	LEED (mg)	Deviant perceptual characteristics
01	M	60	29	12	Levodopa	400	Monopitch, mild hypophonia, short rushes
02	M	65	18	14	ApoLevocarb	1,200	Monopitch, moderate hypophonia, imprecise consonants
03	M	65	23	12	Levodopa	532	Repeated phonemes, imprecise consonants, short rushes
04	M	66	28	35	Levodopa	NA	Harsh voice, monopitch, short rushes, imprecise consonants
05	M	73	27	7	Levodopa	NA	Hypophonia, short phrases, short rushes
06	F	67	30	10	Levodopa, Mirapex	700	Short rushes, fast rate, breathy voice
07	M	72	29	9	Levodopa, amantadine	NA	Imprecise consonants, breathy voice, increased pitch
08	M	85	24	4	Levodopa	400	Harsh voice, imprecise consonants, short rushes
09	M	56	28	25	Levodopa, amantadine	NA	Strained-strangled voice, imprecise consonants, short rushes of speech, phoneme repetitions
10	M	71	25	5	Levodopa	800	Imprecise consonants, distorted vowels, high pitch, hyponasality
11	M	68	25	8.5	Pramipexole, levodopa	300	Strained voice, hoarse voice, hypophonia
12	M	72	24	15	Levodopa, pramipexole	1,300	Hypernasality, monopitch, low pitch
13	M	62	26	3	ApoLevocarb	800	Hoarse voice, imprecise consonants, short rushes
14	M	90	24	10	NA	NA	Hypernasality, high pitch, imprecise consonants, harsh voice
15	M	70	28	2	Levodopa	900	Moderate hypophonia, short rushes, imprecise consonants, high pitch
16	M	73	23	10	Levodopa	800	Moderate hypophonia, hoarse voice, imprecise consonants, monopitch
17	F	71	26	5	Levodopa	NA	Hoarse voice
18	M	64	28	6	Levodopa	600	Imprecise consonants, short rushes, monopitch, moderate hypophonia
19	F	68	28	18	Duodopa	NA	Mild hypophonia, breathy voice, imprecise consonants, short rushes
20	F	73	25	30	Levodopa, Mirapex, amantadine, Apo-gabapentine	1,200	Imprecise consonants, short rushes, audible inhalations
21	M	64	28	8	Mirapex	450	Mild hypophonia, monopitch, imprecise consonants
22	M	71	25	10	Levodopa, pramipexole	900	Imprecise consonants, harsh voice

PD, Parkinson’s disease; MoCA, Montreal Cognitive Assessment; LEED, levodopa equivalent daily dose. One PD-Med participant (PD14) was unsure of their current medication list, which is listed here as NA. Deviant perceptual characteristics for the PD groups correspond to features noted during the habitual monolog speech samples.

**TABLE 2:** Participant demographics for the PD-DBS speaker group.

ID	Sex	Age	MoCA	Years post-diagnosis	Years since DBS surgery	PD medications	LEED (mg)	Deviant perceptual characteristics
01	M	60	24	12	2	Levodopa, amantadine	300	Hoarse, breathy voice, monopitch, imprecise consonants, prolonged intervals
02	F	71	16	25	9	Levodopa	50	Hoarse, breathy voice, imprecise consonants, short rushes, fast rate
03	M	63	24	18	9	Amantadine, levodopa	430	Mild hypophonia, imprecise consonants, short rushes, high pitch
04	M	73	20	12	4	Levodopa	NA	Strained-strangled voice, imprecise consonants, prolonged phonemes, slow rate
05	M	56	27	16	6	Levodopa	NA	Harsh voice, imprecise consonants
06	M	59	16	13	5	Levodopa, amantadine, Sinemet	NA	Mild hypophonia, imprecise consonants, high pitch
07	F	69	25	16	3	Levodopa	550	Moderate hypophonia, strained-strangled voice, audible inspiration, voice breaks
08	M	66	28	14	6	Levodopa	NA	mild hypophonia, strained-strangled voice, pitch breaks, imprecise consonants
09	M	55	28	8	1	Levodopa	500	Imprecise consonants, hoarse voice, short rushes, fast rate
10	M	66	23	4	3	Levodopa	150	High pitch, hypernasality, imprecise consonants, short rushes
11	M	60	25	12	4	Levodopa, ropinirole	NA	Harsh, breathy voice, imprecise consonants, audible inspirations
12	M	66	28	14	7	Levodopa	500	Mild hypophonia, imprecise consonants, short rushes, fast rate
13	M	72	22	15	4	Levodopa	600	Imprecise consonants, breathy voice

PD, Parkinson’s disease; DBS, deep brain stimulation; MoCA, Montreal Cognitive Assessment; LEED, levodopa equivalent daily dose. Deviant perceptual characteristics for the PD groups correspond to features noted during the habitual monolog speech samples.

All PD participants were recruited from the Movement Disorders Centre at University Hospital in London, Ontario (clinic director: MJ). Both groups of PD participants were eligible if they had (a) had received a PD diagnosis by a movement disorders neurologist at least year prior and (b) were stabilized on anti-parkinsonian medication and/or surgical STN-DBS settings. PD-Med participants were also required to have been identified as having at least mild speech impairment, as noted on the Unified Parkinson’s Disease Rating Scale (Part III, speech subsection) in their patient chart history. Due to the smaller and more variable nature of speech outcomes in STN-DBS (Aldridge et al., 2016), PD-DBS participants were not specifically recruited on the basis of the presence of speech impairment and instead reflected a convenience sample. However, all PD-DBS participants did present with at least mild dysarthria. Deviant perceptual characteristics for all PD participants are listed in Tables 1, 2 and were determined by consensus by the first two authors (TK, SA).

All participants were native or near-native speakers of North American English. Hearing and cognitive status were not exclusion criteria for this study, though all but the younger control participants underwent screening for both. All OC and PD participants underwent a hearing screening at 40 dB HL at 500, 1,000, 2,000, and 4,000 Hz or wore hearing aids (2 OC, 5 PD-Med, 3 PD-DBS). YC participants self-reported normal hearing. OC and PD participants also completed the Montreal Cognitive Assessment (MoCA) (Nasreddine et al., 2005). PD participants scores are presented in Tables 1, 2. All but three OC participants scored above 26/30, the suggested cutoff for mild cognitive impairment. Two OC speakers received a score of 25 and one received a score of 21, which is representative of mild cognitive impairment in the aging population (Petersen et al., 2010). Eight participants reported wearing dentures (2 OC, 4 PD-Med, 2 PD-DBS).

### 2.2 Speech task and audio recording procedure

As part of a larger study, all talkers read aloud standardized sentences in seven rate conditions from very slow to very fast, described in more detail in Knowles et al. (2021a). The PD groups participated at a time of day when they would be in their optimal “on” state relative to their PD medications, and all PD-DBS speakers participated with stimulation on and using their standard settings. All participants began the experiment using their habitual speech rate. Three slower speech and three faster speech conditions were then elicited in blocks, with the order of rate manipulation direction counterbalanced across participants. Within each block, three rates were elicited in order of increasing or decreasing speed via magnitude production. For example, within the slower block, participants were asked to complete speech tasks at a rate that felt two times slower, followed by three times and then four times slower than what felt like their normal rate of speaking. Within the fast block, participants spoke at rates that they judged to be two, three, and four times faster than their normal rate of speaking. Magnitude production, rather than a more rigid rate modification technique such as pacing, was used in order to elicit more natural speech (Adams et al., 1993; Turner et al., 1995; Tjaden and Wilding, 2004) that varied across a wide continuum of possible rates for each talker. Actual speech rate was then later calculated in words per minute for each utterance and subsequently transformed into a rate that reflected each individual’s proportional rate relative to their own baseline (below). Participants practiced each new speaking rate using a probe sentence in order to become comfortable using each new rate. The researcher monitored and recorded these practice sessions in order to ensure that, regardless of the actual rate, they were indeed speaking more slowly or quickly relative to the previous condition, as appropriate. All practice utterances were recorded, and the researcher selected one to be used as a model sentence. This model sentence was selected on the basis that it reflected an appropriate relative rate and was representative of the participant’s speech. This model utterance was then played back to them approximately every 10 trials to provide a target for maintaining their target rate throughout the block, with verbal reminders provided by the researcher as needed. The goal of this procedure was to elicit a broad, naturalistic range of rates via an individual’s own psychophysical self-scaling (with supports in place), rather than to elicit specific rate targets.

Instructions for modified speaking rates:

Habitual (1): “Please say the following at your normal speaking rate.”

Slower conditions (3): “Please say the following at a rate that feels like 2×/3×/4× slower than your normal speaking rate. Try to slow your speech down by stretching out your voice, rather than pausing in between words.”

Faster conditions (3): “Please say the following at a rate that feels like 2×/3×/4× faster than your normal speaking rate, while trying to be as accurate as possible.”

Sentences included a randomized set of six sentences per rate condition per participant. Sentences were 5 to 10 words in length (one of each length per condition) randomly selected from the speech intelligibility test (Yorkston et al., 1996). Participants saw three sentences at a time, which were randomly presented with other stimuli as part of the larger study.

All speech tasks were recorded in an audiometric booth (Industrial Acoustic Company) using a 2017 15-in. Dell laptop computer (Inspiron 15). Participants wore an AKG c520 headset microphone positioned 6 cm from the mouth, which was connected to the laptop via a USB preamplifier and digitizing unit (M-Audio MobilePre). Actual speech intensity was calculated by recording participants producing three sustained vowels at approximately 70 dB SPL with a sound level meter positioned 15 cm from their mouth (SPL-A, slow setting), following Dykstra et al. (2015). This resulted in an average calibration factor in dB that was linearly applied to the intensity of each participant’s utterances in subsequent analyses. Utterances were randomized, presented, recorded, and saved via a customized MATLAB script (Version 9.4.0 [R2018a], 2018). Recordings were digitized at 44.1 kHz and 16 bits.

### 2.3 Acoustic analyses

Utterances were later manually checked for any recording errors or major speech disruptions. Less than 5% of utterances were excluded at this stage (within each group this corresponded to YC: 2%; OC: 2%; PD-Med: 3%; PD-DBS: 10%). Utterances were then manually segmented at the utterance boundaries to remove initial and trailing silences by the first author using a custom Praat script (Boersma and Weenink, 2021). A maximum of 42 utterances per participant were possible (6 sentences × 7 rates). Rate was calculated in words per minute (WPM) by dividing the number of words in each utterance by the utterance duration. Each participant’s baseline habitual speaking rate was calculated based on their average speech rate in the habitual condition (as in Knowles et al., 2021b). All utterances were then transformed into a proportion of this rate. All utterances with proportional rates less than or greater than 1 were produced at a slower- or faster-than-habitual speech rate, respectively, for each individual. For example, if a speaker had a mean habitual rate of 200 WPM, a sentence they produced at 300 WPM would have a proportional rate of 1.5.

Three acoustic measures relating to voice production were chosen for their sensitivity to voice changes in PD and their ability to measure voice production in continuous speech. These included speech intensity, smoothed cepstral peak prominence (CPP), and *f*_0_. CPP and *f*_0_ were measured using an adapted version of the batch CPP Praat plugin described in Heller Murray et al. (2022). Minimum and maximum peak searches were set to 60 Hz and 330 Hz, respectively. CPP was extracted from only voiced portions of the sound using the “voice detection” approach described in Heller Murray et al. (2022). *f*_0_ was extracted from the full utterance. Speech intensity was extracted from the utterances using another script that automatically removed silent portions from the signal using the Trim Silences function in Praat (threshold: −35 dB; minimum silence duration of 100 ms) (Boersma and Weenink, 2021). Speech intensity was then calibrated using the calibration factor described above.

### 2.4 Statistical analyses

Habitual rate and categorical rate differences for the sentence production task are described and reported in Knowles et al. (2021a). We briefly summarize these previous findings in the results and report group differences of proportional rate production. All outcomes in the present study were measured using linear mixed effects regression models.

Two separate models were run for each of the three acoustic outcomes: one to examine the effect of slower speech, and one of faster speech (six models in total). Distinct models for the two rate modification directions were chosen in order to characterize and more easily interpret patterns at relatively slower and faster rates, which reflect distinct psychophysical goals (following Knowles et al., 2021b). This aids in interpretation of clinical findings as well, as slower but not faster rates are often selected as speech therapy goals for dysarthria. Slower-speech models included all utterances with a proportional rate less than or equal to 1, and faster-speech models included proportional rates greater than 1. Rate was calculated separately for each utterance.

Each acoustic outcome was modeled as a function of speaker group, proportional rate of speech, and their interaction. Speaker sex and sentence length were included as covariates. Speaker group was coded using reverse Helmert contrasts, such that the first level contrast can be interpreted as comparing the YC group to the mean of the OC and both PD groups (YC = +3/4; Others = −1/4), the second contrast compares the OC group to the mean of the combined PD groups (OC = +2/3; PD-Med = −1/3; PD-DBS = −1/3), and the third level contrast compares the estimated means of the two PD groups (PD-Med = +1/2; PD-DBS = −1/2). Proportional rate and sentence length were entered as continuous predictor variables, and speaker sex was sum coded (Female = +1; Male = −1). Where possible, random effects terms included by-participant intercepts and slopes for proportional rate, and by-item intercepts. Random effects structures were simplified as needed if singular model fits were observed. All model residuals were checked and met assumptions of normality and homoscedasticity.

In the case of group by rate interactions, pairwise comparisons for each group were run using the emmeans package (Lenth, 2023) that compared changes in each acoustic measure across the range of rate modifications specified in the model. Lastly, a series of repeated measures correlations were used to explore how the three voice measures of interest patterned together across the dataset.

## 3 Results

### 3.1 Speaking rate adjustments

**FIGURE 1:**
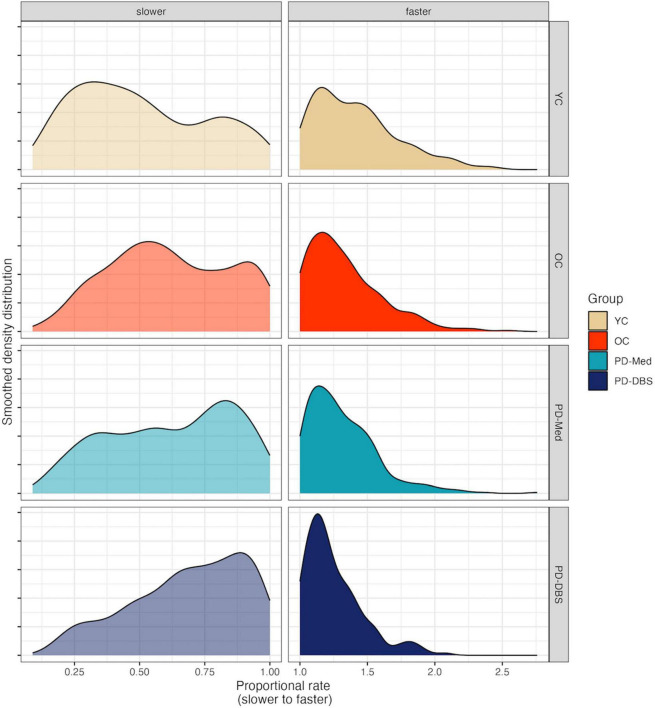
Smoothed density plots for proportional speech rate adjustments for each speaker group. YC, younger controls; OC, older controls; PD-Med, Parkinson group on standard levodopa medication; PD-DBS, Parkinson group with deep brain stimulation.

Speech rate adjustments for sentence production have been reported in Knowles et al. (2021a) in actual WPM and proportional speech rate. Briefly, there were no statistical differences in actual habitual WPM for any of the groups. While there was variability in the magnitude of rate variation in the slower and faster rate conditions across groups, speech rate did vary in the expected directions across all rate conditions. The greatest magnitude of change was found for the YC group and the smallest magnitude of change was found for the PD-DBS group. Proportional rate adjustments for each group appear in Figure 1.

### 3.2 Speech intensity

Model output for speech intensity appears in Table 3. In the slow speech model, younger controls were found to produce an overall speech intensity 5.27 dB SPL higher than the average of the other three groups (CI : [2.35, 8.19], *p* < 0.001). Younger participants also increased their speech intensity as their speech rate slowed, while the other groups did not, as evidenced by a significant interaction between speaker group and speech rate for the young vs. old contrast (estimate: –5.97 dB SPL; CI : [–8.90, –3.04]; *p* < 0.001).

**TABLE 3:** Coefficients table for the speech intensity models.

	Slower speech			Faster speech		
**Predictors**	**Estimates**	**CI**	***p***	**Estimates**	**CI**	***p***
(Intercept)	73.64	71.10 to 76.19	**< 0.001**	73.57	71.30 to 75.83	**< 0.001**
groupYCvRest	5.27	2.35 to 8.19	**0.001**	1.30	−0.96 to 3.57	0.252
groupOCvPD	1.98	−1.22 to 5.18	0.222	1.28	−1.32 to 3.89	0.329
groupPD-MedvPD-DBS	2.63	−1.14 to 6.41	0.169	−1.78	−4.90 to 1.34	0.259
prop_rate	0.01	−1.41 to 1.42	0.994	0.75	−0.05 to 1.55	0.067
sexFvM	−0.37	−1.12 to 0.38	0.326	−0.29	−1.16 to 0.58	0.506
nwords	−0.13	−0.41 to 0.16	0.371	−0.15	−0.41 to 0.11	0.232
groupYCvRest × prop_rate	−5.97	−8.90 to −3.04	**< 0.001**	−0.86	−2.48 to 0.75	0.286
groupOCvPD × prop_rate	−0.91	−4.12 to 2.30	0.574	−0.42	−2.34 to 1.51	0.665
groupPD-MedvPD-DBS × prop_rate	−4.07	−7.89 to −0.25	**0.037**	0.52	−1.82 to 2.86	0.659
**Random effects**						
σ^2^	2.67			2.72		
τ_00_	25.22 _*participant*_			9.98 _*participant*_		
	2.20 _*item*_			1.38 _*item*_		
τ_11_	23.80 _*participant.prop–rate*_			5.00 _*participant.prop*–rate_		
ρ_01_	−0.84 _*participant*_			−0.35 _*participant*_		
ICC	0.82			0.84		
*N*	67 _*participant*_			67 _*participant*_		
	42 _*item*_			39 _*item*_		
Observations	1,504			1,171		
Marginal *R*^2^/conditional *R*^2^	0.088/0.840			0.026/0.841		

Bold values mean statistically significant results (*p* < 0.05).

Conversely, a significant interaction for the PD-Med vs. PD-DBS group and speech rate indicated that talkers with DBS decreased their speech intensity at slower rates compared to the PD-Med group (estimate: –4.07 dB SPL; CI : [–7.89, –0.25]; *p* = 0.037). No significant interaction was found between the OC group and the PD groups and rate of speech in the slow speech model.

Significant changes in speech intensity in the YC and PD-DBS group at slow rates were confirmed in *post-hoc* pairwise analyses. Across the range of speech rate modifications, the YC group increased their speech intensity by an average of 4.07 dB SPL (*p* = 0.001), and the PD-DBS group decreased their intensity by −3.493 dB SPL (*p* = 0.017). No significant differences were observed within the OC or PD-Med groups.

**FIGURE 2:**
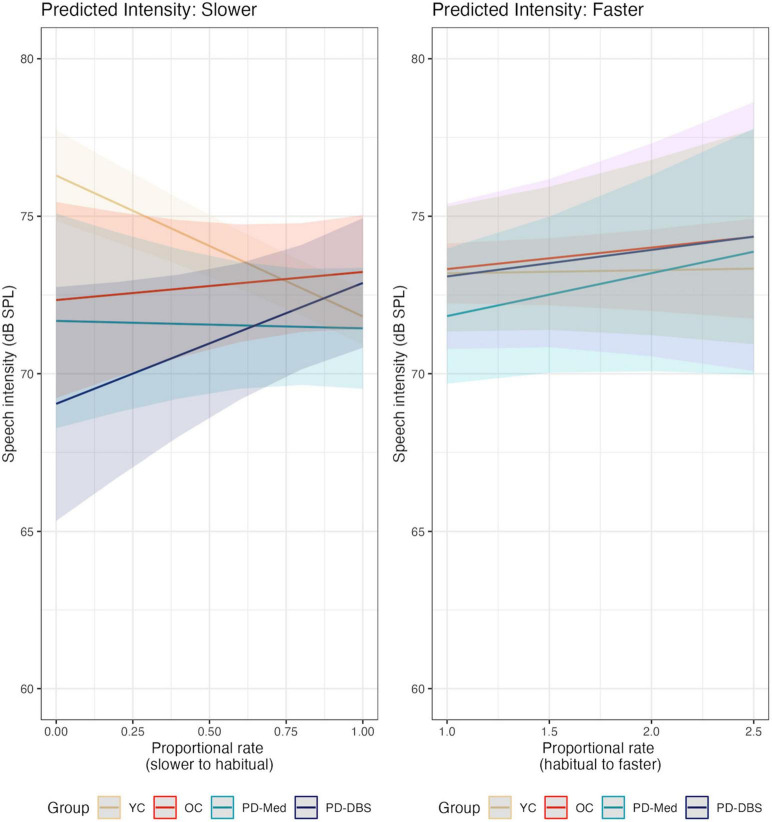
Model predictions for speech intensity as a function of proportional speech rate and speaker group.

**FIGURE 3:**
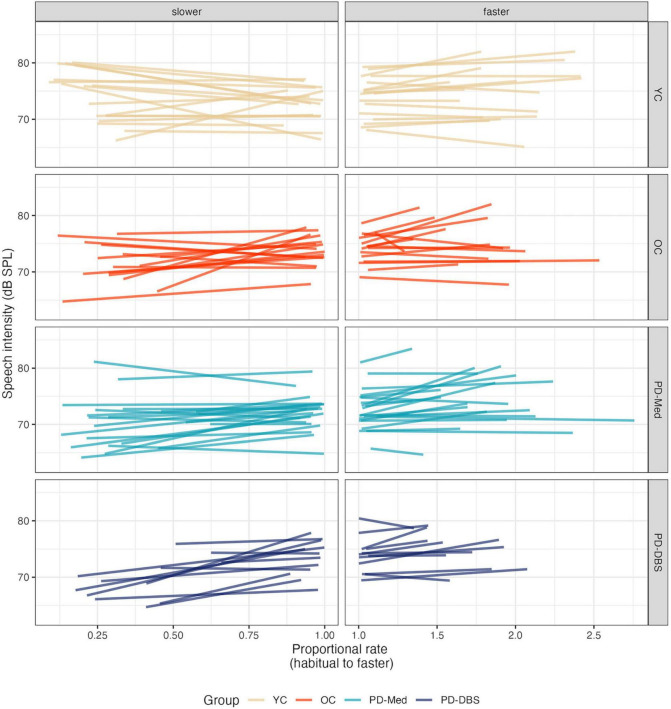
Empirical plots for speech intensity as a function of proportional speech rate and speaker group. Individual lines represent individual speaker means.

No significant main effects nor interactions were found for speech intensity in the fast speech model. However, Figure 2 shows that, despite substantial variability and a lack of an overall group effect, there was an overall trend for increased speech intensity at faster rates. Figure 3 presents the empirical trendlines for each participant, showing that most but not all participants demonstrated this pattern, to varying degrees.

### 3.3 Cepstral peak prominence

Regarding CPP, a similar pattern to that of speech intensity was found in slow speech; model outcomes appear in Table 4. Namely, younger speakers demonstrated higher overall CPP and higher CPP at slower rates, while the PD-DBS group demonstrated a decline in CPP at slower rates. The main effect of the young versus old contrast found CPP to be, on average, 5.05 dB higher for the YC group compared to the others (CI : [3.22, 6.89]; *p* < 0.001). A significant group by speech rate interaction for the young versus old contrast also suggested that the younger speakers produced higher CPP values in slow speech while the other groups did not (estimate: –5.07 [–6.83, –3.31]; *p* < 0.001). Non-significant trends emerged for the OC versus PD and PD-Med versus PD-DBS contrasts, suggesting an overall pattern of higher CPP for YC > OC > PD-Med > PD-DBS (OC vs. PD–estimate: 1.73 [–0.28, 3.74]; *p* = 0.09; PD-Med vs. PD-DBS–estimate: 2.23 [–0.15, 4.60]; *p* = 0.07).

**TABLE 4:** Coefficients table for the cepstral peak prominence models.

	Slower speech			Faster speech		
**Predictors**	**Estimates**	**CI**	***p***	**Estimates**	**CI**	***p***
(Intercept)	10.92	9.74 to 12.09	**< 0.001**	10.19	9.36 to 11.01	**< 0.001**
groupYCvRest	5.05	3.22 to 6.89	**< 0.001**	1.47	0.03 to 2.92	**0.046**
groupOCvPD	1.73	−0.28 to 3.74	0.091	1.48	−0.20 to 3.16	0.083
groupPD-MedvPD-DBS	2.23	−0.15 to 4.60	0.066	0.18	−1.85 to 2.20	0.864
prop_rate	−0.33	−1.18 to 0.52	0.444	0.55	0.09 to 1.00	**0.020**
sexFvM	0.38	−0.07 to 0.82	0.094	0.33	−0.11 to 0.76	0.138
nwords	0.02	−0.09 to 0.12	0.761	0.03	−0.03 to 0.09	0.361
groupYCvRest × prop_rate	−5.07	−6.83 to −3.31	**< 0.001**	−0.84	−1.75 to 0.06	0.068
groupOCvPD × prop_rate	−1.53	−3.47 to 0.40	0.119	−1.04	−2.15 to 0.07	0.067
groupPD-MedvPD-DBS × prop_rate	−2.93	−5.24 to −0.62	**0.014**	−0.45	−1.83 to 0.94	0.522
**Random effects**						
σ^2^	1.19			1.42		
τ_00_	9.88 _*participant*_			3.75 _*participant*_		
	0.27 _*item*_			0.03 _*item_cond*_		
τ_11_	8.38 _*participant.*_ _*prop*_*rate*_			1.07 _*participant.prop*_*rate*_		
ρ_01_	−0.86 _*participant*_			−0.59 _*participant*_		
ICC	0.78			0.65		
*N*	67 _*participant*_			67 _*participant*_		
	42 _*item*_			39 _*item*_		
Observations	1,504			1,171		
Marginal *R*^2^/conditional *R*^2^	0.239/0.832			0.051/0.666		

Bold values mean statistically significant results (*p* < 0.05).

A significant interaction between the two PD groups and rate of speech demonstrated that the PD-DBS speakers’ CPP values significantly decreased with slower rates (estimate: –2.93 [–5.24, –0.62]; *p* = 0.014).

Pairwise comparisons confirmed these patterns. The YC group demonstrated an increase in CPP by 3.761 dB (*p* < 0.001) and the PD-DBS group showed a decrease of −2.654 dB (*p* = 0.003). No significant change was found within the OC or PD-Med groups.

**FIGURE 4:**
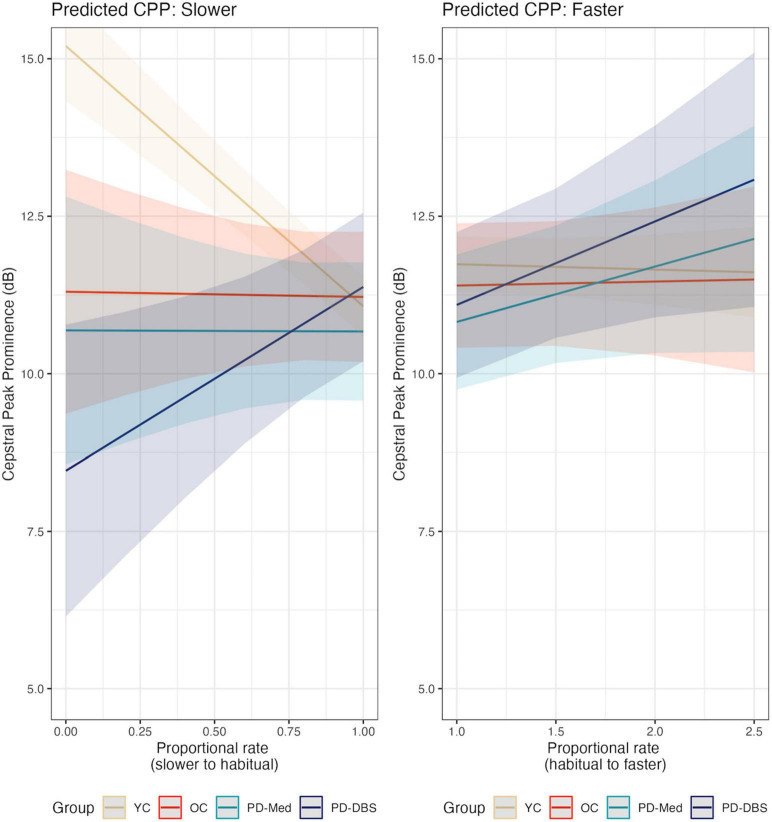
Model predictions for cepstral peak prominence as a function of proportional speech rate and speaker group.

**FIGURE 5:**
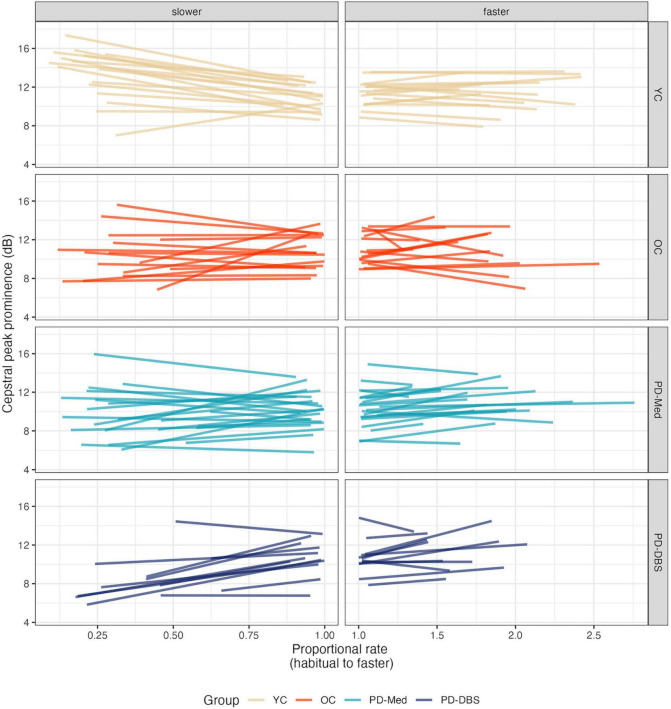
Empirical plots for cepstral peak prominence as a function of proportional speech rate and speaker group. Individual lines represent individual speaker means.

In fast speech, a different pattern emerged. Once again the YC group demonstrated overall higher CPP than the other groups, captured by a main effect for the young versus old contrast (estimate: 1.47 [0.03, 2.92]; *p* = 0.046). An overall main effect of speech rate was also found, indicating that, on average, across all groups, there was an overall increase in CPP values as speech rate increased (estimate: 0.55 dB [0.03, 2.92]; *p* = 0.046). Non-significant interactions with rate of speech and speaker group indicate that this was largely driven by the two PD groups, as can be seen in Figure 4 (YC versus Rest–estimate: –0.84 [–1.75, 0.06]; *p* = 0.07; OC versus PD–estimate: –1.04 [–2.15, 0.07]; *p* = 0.07). Figure 5 shows empirical data for CPP for all speakers.

### 3.4 *f*_0_

Model output for *f*_0_ appears in Table 5. Overall, in slow speech, the OC group produced a lower *f*_0_ compared to the other groups, captured by a main effect of the OC versus PD groups contrast (estimate: –24.19 [–41.85, –6.52]; *p* = 0.008). This was confirmed by pairwise comparisons, which showed that the OC group decreased their *f*_0_ by an average of −16.16 Hz across the range of speech rates (*p* = 0.007). No significant change in *f*_0_ was observed within any of the other groups. No other main effects for the other group contrasts were found. A main effect of speech rate indicated that, overall, speakers produced a lower pitch at slower rates (estimate: 7.74 Hz [1.07, 14.40]; *p* = 0.024). A predictable main effect of sex was also found; females spoke on average 26.69 Hz higher than males (CI : [21.75, 31.63]; *p* < 0.001).

**TABLE 5:** Coefficients table for the *f*_0_ models.

	Slower speech			Faster speech		
**Predictors**	**Estimates**	**CI**	***p***	**Estimates**	**CI**	***p***
(Intercept)	157.12	148.52 to 165.73	**< 0.001**	163.64	151.93 to 175.35	**< 0.001**
groupYCvRest	9.14	−6.76 to 25.05	0.255	15.15	−2.98 to 33.27	0.099
groupOCvPD	−24.19	−41.85 to −6.52	**0.008**	−17.27	−38.59 to 4.05	0.110
groupPD-MedvPD-DBS	10.48	−10.62 to 31.57	0.325	20.42	−5.66 to 46.50	0.123
prop_rate	7.74	1.07 to 14.40	**0.024**	0.06	−6.16 to 6.28	0.984
sexFvM	26.69	21.75 to 31.63	**< 0.001**	28.25	23.44 to 33.05	**< 0.001**
nwords	−0.50	−1.10 to 0.11	0.099	−0.39	−1.41 to 0.64	0.443
groupYCvRest × prop_rate	2.65	−11.41 to 16.70	0.708	−4.38	−16.43 to 7.66	0.467
groupOCvPD × prop_rate	16.02	0.25 to 31.79	**0.047**	7.57	−7.39 to 22.53	0.315
groupPD-MedvPD-DBS × prop_rate	5.91	−13.34 to 25.17	0.542	−5.81	−24.58 to 12.95	0.539
**Random Effects**						
σ^2^	263.78			296.10		
τ_00_	675.57 _*participant*_			461.11 _*participant*_		
	1.70 _*item*_			11.65 _*item*_		
τ_11_	354.37 _*participant.prop_rate*_			155.12 _*participant.prop_rate*_		
ρ_01_	−0.76 _*participant*_			−0.62 _*participant*_		
ICC	0.59			0.52		
*N*	67 _*participant*_			67 _*participant*_		
	42 _*item*_			39 _*item*_		
Observations	1,504			1,171		
Marginal *R*^2^/conditional *R*^2^	0.520/0.803			0.548/0.782		

Bold values mean statistically significant results (*p* < 0.05).

**FIGURE 6:**
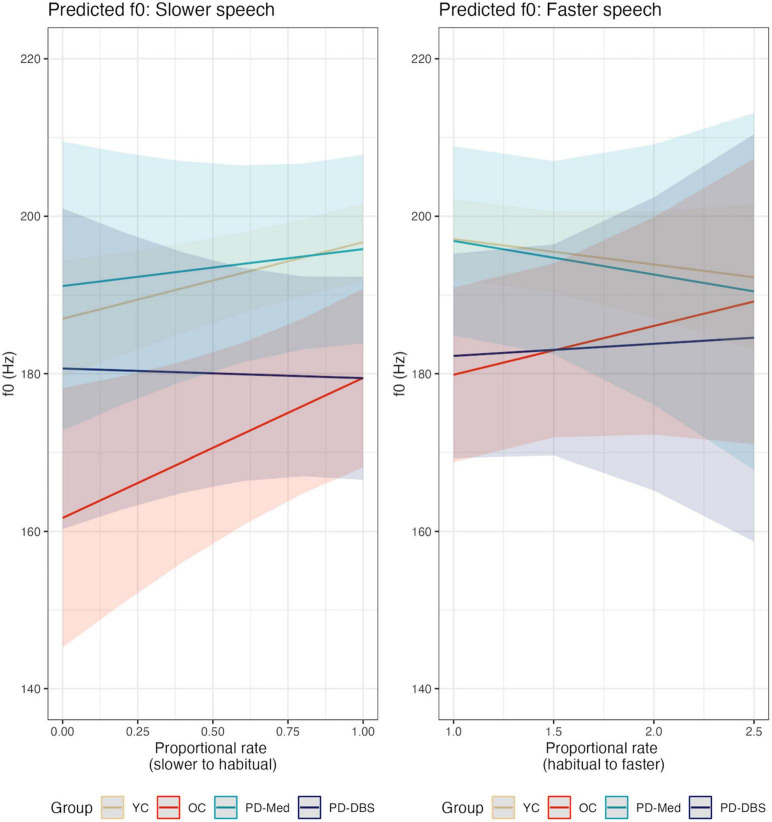
Model predictions for *f*_0_ as a function of proportional speech rate and speaker group.

**FIGURE 7:**
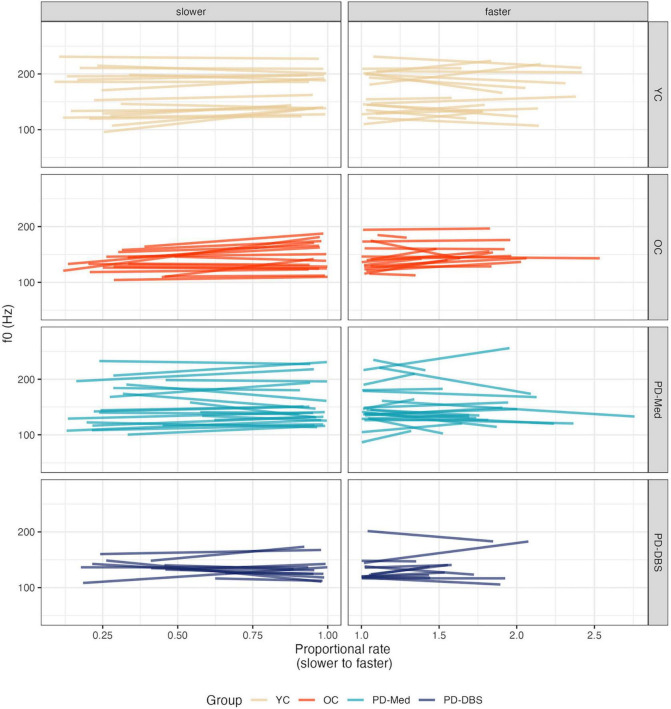
Empirical plots for *f*_0_ as a function of proportional speech rate and speaker group. Individual lines represent individual speaker means.

An interaction between rate of speech and the OC versus PD group contrast indicated that, not only did the older controls speak at an overall lower pitch, but lowered their pitch to a greater extent in slow speech compared to the other groups (estimate: 16.02 [0.25, 31.79]; *p* = 0.047). This is evident in Figure 6. Figure 7 shows empirical data for *f*_0_ for all speakers.

The only significant effect in the fast speech model was for speaker sex (females spoke with an *f*_0_ 28.25 Hz higher than males; CI : [23.44, 33.05] *p* < 0.001).

### 3.5 Relationships between acoustic measures of voice

Speech intensity and CPP exhibited a moderate-to-strong positive correlation (repeated measures coefficient: *r* = 0.63 CI: [0.60, 0.65], *p* < 0.001). Speech intensity and *f*_0_, on the other hand, showed a very weak positive correlation (repeated measures coefficient: *r* = 0.06 CI: [0.03, 0.10], *p* = 0.001). CPP and *f*_0_ demonstrated a very weak negative correlation (repeated measures coefficient: *r* = −0.11 CI: [−0.15, −0.07], *p* < 0.001).

## 4 Discussion

The primary purpose of this study was to explore how phonatory acoustics change as a function of speech rate modifications across a broad range of both slower and faster rates of speech in people with and without PD. While speech rate modifications as a dysarthria management approach are often recommended in order for speakers to more easily produce more canonical articulatory positions, the results of the current study demonstrate that there may be consequences on other speech subsystems that warrant consideration. Indeed, adjustments to speaking rate invoke changes across articulatory, phonatory, and respiratory systems (Dromey and Ramig, 1998), though not all these changes may be beneficial for all individuals. Overall, in the present study, slower-than-habitual rates of speech were associated with differential effects on measures of phonation across speaker groups, with the most extreme patterns observed for the young, healthy group and the PD-DBS talkers. All talkers spoke at a lower pitch at slower rates. Young healthy talkers spoke louder and with improved voice quality while talkers in the PD-DBS group spoke more quietly and with poorer voice quality. The older control group and the PD-Med group behaved in between these two extremes. At faster rates of speech, all groups uniformly improved their voice quality, but no other significant changes in speech intensity or pitch were observed. Results are first discussed in terms of the acoustic outcomes, then contextualized in theories of speech motor control.

### 4.1 Changes in phonatory acoustics as a function of speech rate

A small body of previous literature has reported on changes in speech intensity as a function of rate. For example, Tjaden and Wilding (2004) found that speaking at a (single) slower rate was associated with a ∼1 dB SPL increase in speech intensity for people with PD. Conversely, Dromey and Ramig (1998) found that, in a small cohort of young healthy talkers, speech rate was positively associated with speech intensity, such that faster but not slower rates were associated with intensity increases. Specifically, slower speech (elicited in two slower speech conditions) resulted in lower speech intensity, while faster speech resulted in greater speech intensity. The present study found that the younger controls did increase their speech intensity at slower rates (consistent with Tjaden and Wilding, 2004), but the other groups did not. In fact, the PD-DBS group produced lower speech intensity at slower rates, consistent with Dromey and Ramig (1998). In the present study, faster speech rates were not associated with significant changes in speech intensity, counter to what some authors have found previously in healthy talkers (Dromey and Ramig, 1998; Wohlert and Hammen, 2000) and in a case study of a talker with dysarthria secondary to traumatic brain injury (D’Innocenzo et al., 2006). However, in the present results, a non-significant trend for increased intensity at faster rates showed that, despite substantial interspeaker variability, some speakers did demonstrate this pattern. Differences here with past literature could be due in part to the task; here, speakers read aloud sentences ranging from 5 to 10 words in length compared to repeating a single sentence multiple times (Dromey and Ramig, 1998; Wohlert and Hammen, 2000)^1^.

With regards to voice quality acoustic measures, CPP has recently been favored over more traditional measures of phonatory perturbation measures such as jitter and shimmer (Patel et al., 2018). CPP reflects the relationship of periodic to aperiodic energy in a signal and has become a popular index of dysphonia, especially in connected speech. It is also closely associated with speech intensity. Previous research in PD has shown that increased CPP can capture positive post-treatment vocal quality change following LSVT-LOUD (Alharbi et al., 2019), which indexes improved harmonic structure. In the present study, the PD-DBS group produced lower CPP values at slower rates, indicating potentially poorer vocal control. Conversely, the younger healthy talkers produced higher CPP values as they slowed their rate of speech. Taken with the speech intensity findings, this reflects two very different consequences of rate reduction on phonatory control. If rate reduction were associated with greater glottal control, overall increases in CPP would be observed, such as was the case for the YC speakers. However, the decrease observed for the PD-DBS group may actually reflect poorer glottal closure and control. Decreases in acoustic voice quality are also a marker of aging [e.g., harmonics-to-noise ratio; Ferrand (2002)]. The younger talkers may have been able to exercise greater control over a more stable vocal system compared to the older adults in general. It could be the case that slight increases or decreases to laryngeal resistance impacted the speaker groups in the present study differently, too. For example, slight increases in resistance may be associated with limited change in voice quality in an unimpaired speaker, but worse voice quality in a speaker with impaired vocal control. While the relationship with speech rate was found to be stronger for CPP than for speech intensity, the moderate-to-strong relationship between CPP and intensity affirm that these changes, driven in part by the degree of glottal closure, pattern together.

Overall, all groups tended to decrease their *f*_0_ at slower rates, though this was only found to be significant for the older neurologically healthy control group. Dromey and Ramig (1998) found that *f*_0_, in healthy male talkers, did change as a function of rate, but found that this typically supported higher overall *f*_0_ at faster rates of speech rather than a clear change at slower-than-normal rates. However, Dromey and Ramig (1998) also found that *f*_0_ variability decreased at slower rates, consistent with perceptual accounts of slow speech sounding monotonous. Little to no relationship was found between *f*_0_ and speech intensity or CPP overall, suggesting that these adjustments are occurring independently of one another, at least when mediated by speech rate control. It is worth nothing that the present study looked at mean values extracted across the duration of an utterance in order to characterize the overall acoustic changes that occurred during speech rate adjustments. Fluctuations within an utterance were not taken into account. However, it is likely that prosodic changes in speech intensity and *f*_0_ also occurred as talkers modified their speaking rate. The extent to which these within-utterance prosodic changes that occur in speech rate modification impact auditory-perceptual outcomes such as speech naturalness remain an open question for future study.

### 4.2 Implications for speech motor control

Adams et al. (1993) suggested that there may be distinct speech motor control strategies when speakers decrease or increase their speech rate. Slow speech may involve multiple submovements across individual speech segments that are subject to neural feedback mechanisms, whereas fast speech may involve single, discrete movements to produce individual gestures. Under this interpretation, these findings have implications for neural models of speech sensorimotor control and the role of feedback versus feedforward neural control processes involved in speech rate modification. According to the widely used Directions of Velocities of Articulators (DIVA; Guenther, 2006, 2016), before producing an utterance, a motor command for speech is first neurally encoded and an efference copy of this command is used to predict and incorporate the effects of vocal motor actions. Detection of perceived mismatches between the expected and perceived output that surpass a certain threshold lead to corrections in the motor command. In slow speech, there may be sufficient time for multiple detections and adjustments based on feedback from perception of one’s own speech to occur. If slower speech is comprised of multiple submovements, this gives rise to more opportunities for variability in these adjustments to arise. Conversely, fast speech may be more ballistic in nature and rely more on feedforward mechanisms. A recent study by Abur et al. (2018) found that people with PD, relative to healthy speakers, demonstrated less adaptation and more variability in response to *f*_0_ perturbations. The authors suggested these differences were related to deficits in perceptual adaptation, rather than purely perceptual deficits in perceptual acuity, which were not found to differ across the groups.

One physiological possible explanation for the observed vocal changes is that the PD-DBS talkers achieved a slower rate of speech by decreasing glottal airflow and simultaneously increasing laryngeal resistance in order to conserve respiratory airflow over the course of the utterance. This may have resulted in the acoustic patterns found here, namely, decreased intensity, pitch, and poorer acoustic voice quality (Plant and Younger, 2000). It could also be the case that slow speech places a greater demand on the respiratory system, and lower speech intensity and lower pitch may be a compensatory mechanism used to maintain continuous respiratory output across an utterance during slow speech. A small body of evidence suggests that, while speech intensity is not typically impaired following STN-DBS (Aldridge et al., 2016), there is evidence of impaired respiratory control (Hammer et al., 2010; Chalif et al., 2014). STN-DBS stimulation may be associated with increased speech intensity (Lundgren et al., 2011), which may be driven by excessive glottal closure and respiratory over-drive. Under the increased respiratory demands imposed by slower speech, such characteristics may partially explain the findings observed in the present study of decreased intensity and increased CPP for the talkers with STN-DBS. Other potential contributing factors could be related to effects of utterance length on speech breathing (Sperry and Klich, 1992; Winkworth et al., 1994; Huber, 2008), in particular for older speakers (Huber, 2008).

While the purpose of the task was for speakers to modify their rate, the present results demonstrate the, sometimes detrimental, multisystem changes they were likely enacting to achieve this. The results of Knowles et al. (2021a) showed that the PD-DBS speakers nevertheless were judged to be more intelligible at slower rates, despite the decreases in speech intensity and voice quality reported here. Curiously, these same speakers also did not show clearly improved vowel space or stop distinctiveness at slower speech (in a nonsense word carrier phrase task) (Knowles et al., 2021b), indicating that improvements in intelligibility may be attributable to other factors. Time for the listener to parse speech, rather than improvements in speech clarity or audibility, may be especially important for more severe speakers, for example.

### 4.3 Limitations

The findings presented here should be considered in the context of study limitations. There was substantial variability amongst speakers, including individuals with hearing aids and mild cognitive impairment in the clinical groups. Relatively lenient inclusion criteria were chosen intentionally to include a representative sample of speakers with speech impairments, though this variability limits the generalizability of this study’s results. Another consideration is that the rate modifications presented here represent a broad spectrum of speech rate adjustments and without much training. In a clinical context, care would ideally be undertaken to ensure an individual could produce a target rate effectively. While the results here point to multisystem adjustments that occur with speaking rate changes, an open question is the extent to which these same changes would be observed after more time was devoted to practicing a given rate over multiple clinical sessions.

### 4.4 Clinical implications and conclusion

Results from this study provide more evidence to suggest that modifying one’s speech rate is associated with multisystem changes. In rate modification, the target change is typically articulatory precision. However, the degree to which other speech subsystems are affected should be considered with caution, as some speech or voice symptoms may actually become more severe. In particular, the PD-DBS group in this study spoke more quietly and with poorer voice quality at slower rates. Given that DBS is often associated with detrimental speech changes above and beyond the typical speech symptoms present in PD (Aldridge et al., 2016), this suggests that some individuals may not benefit from a rate reduction strategy.

## Data availability statement

The raw data supporting the conclusions of this article will be made available by the authors, without undue reservation.

## Ethics statement

The studies involving humans were approved by the Health Sciences Research Ethics Board at Western University and the Lawson Health Research Institute. The studies were conducted in accordance with the local legislation and institutional requirements. The participants provided their written informed consent to participate in this study.

## Author contributions

TK: Conceptualization, Data curation, Formal Analysis, Investigation, Methodology, Writing – original draft, Writing – review & editing. SA: Conceptualization, Writing – review & editing. MJ: Resources, Writing – review & editing.

## Footnotes

1 Note that the case study presented in D’Innocenzo et al. (2006) did use sentence lists, rather than single phrase repetition, so task is likely not the only difference.
